# Case series of autosomal recessive hereditary spastic paraparesis with novel mutation in SPG 7 gene

**DOI:** 10.17712/nsj.2017.4.20170253

**Published:** 2017-10

**Authors:** Shakya Bhattacharjee, Nicholas Beauchamp, Brian E. Murray, Timothy Lynch

**Affiliations:** *From the Department of Neurology (Bhattacharjee), from Plymouth Hospital NHS Trust, Department of Neurology (Bhattacharjee), Plymouth Hospital NHS Trust, from Sheffield Diagnostic Genetics Service (Beauchamp), Sheffield Children’s NHS Foundation Trust, Sheffield, United Kingdom, and from Hermitage Medical Clinic (Murray), Mater University Hospital and Dublin Neurological Institute (Lynch), Dublin, Ireland*

## Abstract

Autosomal recessive hereditary spastic paraparesis is rare.We present 4 patients with slowly progressive predominantly lower limb spasticity and ataxia. Only one patient had family history of ataxia but without any underlying diagnosis. All of them proved negative for the mutation of Spinocerebelalr ataxia genes SCA 1,2,3 and 6. All had mutation in the SPG 7 gene suggestive of autosomal recessive hereditary spastic paraparesis. One of the heterozygous mutatnts showed a novel c1617delC, p(Val540fs) frameshift mutation in exon 12 of the SPG 7 gene. SPG7 mutation accounts for 1.5-7% of all the HSP but it is the cause of undiagnosed ataxia in 18.6% in a recent case series. SPG7 mutation should be remembered as an important cause of undiagnosed ataxia especially where next generation sequencing is not widely avaialbale or affordable.

The spastic paraplegia 7 gene (SPG7) is located in the long (q) arm of chromosome 16 at position 24.3.Protein product of SPG 7 gene Paraplegin is involved in the maintenance of mitochondrial function by forming a part of the mitochondrial multimeric mAAA metaloprotease complex.^1^,^2^ More than 77 different mutations of the SPG7 gene have been described in the literature so far.^3^ Mutation is the SPG7 is responsible for autosomal recessive Hereditary Spastic Paraparesis (ARHSP). Though SPG7 mutation accounts for 1.5-7% of all the HSP but it is the cause of undiagnosed ataxia in 18.6% in a recent case series.^1^,^4^ Spastic paraplegia 7 (SPG-7) can present as a pure and complex phenotype.^3^,^5^ The complex phenotype shows clinical features like younger age of onset, optic nerve involvement, upper limb involvement, cognitive deficits and peripheral neuropathy in addition to features like pyramidal tract signs and ataxia seen in the pure phenotype of Spastic paraplegia.^5^ Many patients with SPG 7 mutation present with subtle signs which can only be diagnosed with careful examination.^4^ The mechanism of neuronal damage in SPG 7 mutation is not yet fully understood.

We described a cohort of 4 patients diagnosed as Autosomal Recessive HSP with a novel mutations of the SPG 7 gene. This mutation is an important cause of undiagnosed ataxia with significant risk of transmission to the future generations. We described not only a probably pathogenic noble SPG 7 gene mutation but also suggested to carefully search for ophthalmological signs like ptosis and ophthalmoparesis as they remain an important diagnostic clue for SPG 7 mutation testing.

## Case Report

### Patient information

All patients in our case series were Caucasian male between 32 to 47 years old when the symptoms started (**Table 1**). However one patient presented to us at the age of 70 years and another at 61 years of age. All of our patients presented with slowly progressive ataxia and 2 had fall on multiple occasions. Only one person had family history of undiagnosed ataxia. All of them were referred by either the GP or the peripheral hospitals as the cause of their symptoms were unclear.

**Table 1:** Timeline table shows demography, clinical features and investigation outcome in 4 SPG 7 positive hereditary spastic paraparesis patients.

Patient characteristics	Patient 1	Patient 2	Patient 3	Patient 4
Gender	Male	Male	Male	Male
Onset (yrs)	32	46	45	47
Age (yrs) at presentation	42	50	70	61
First Review date	12.1.15	27.6.14	15.7.15	14.616
Symptoms at presentation	Slowly progressive ataxia-10years, slurred speech-3 years, pins and needles in left lower limb -2 years	Progressive both calf pain, incoordination and intermittent falls, lower limb weakness	Slowly progressive ataxia for 25 years bilateral ptosis-20 years slurred speech-7 years	Slowly progressive ataxia , frequent falls in last 3 years, intention tremor slurred speech -10 years
Past history	Occasional headache	nil	Prostate Carcinoms	Nil
Family history	nil	nil	Brother and Sister-undiagnosed spasticity	Nil
Clinical signs (1^st^ visit)	Dysarthria, cerebellar ataxia, spastic lower limbs, brisk both KJs, AJs, upgoing plantars, partial external ophthalmoplegia on horizontal gaze	Spastic and broad-based gait, impaired heel-toe walk, downgoing plantars but brisk KJs and AJs, partial external ophthalmoplegia on horizontal gaze, slow saccade	brisk lower limb reflexes, left LL drift, both upgoing plantars, broad based ataxic gait, impaired heel shin test, bilateral asymmetrical ptosis (L>R), partial external horizontal ophthalmoplegia	Both lower limb spasticity, both ankle clonus, brisk lower limb reflexes, absent plantar responses dysarthria, cerebellar ataxia
Patient Concerns	Multiple falls, job related concern, risk of transmission to next generation	Multiple falls, job fitness, Risk of transmission to children	Multiple falls, poor mobility, driving	Multiple falls, poor mobility

### Clinical findings

All of them demonstrated spasticity in the lower limbs. First three patients revealed some degree of horizontal ophthalmoplegia (**Table 1**). The third patient had asymmetric ptosis in addition to ophthalmoplegia. All of them had signs of cerebellar ataxia.

### Diagnostic information

All 4 patients were subject to routine bloods, B12, folate, thyroid function test, copper, nerve conduction studies, etc. Then all had routine genetic testing for ataxia including spinocerebellar ataxia, Friedreich’s ataxia (Frataxin) Fragile X Associated Tremor/Ataxia syndrome (FRXTA) (**Table 2**). They subsequently had next generation sequencing of 21 gene panel for hereditary spastic paraparesis.

**Table 2:** Results of the Magnetic Resonance imaging of the brain, nerve conduction and genetic studies of the patients.

2^nd^ visit	3 months later	4 months later	4 months later	4 months later
MRI brain	Cerebellar atrophy	Cerebellar atrophy	Cerebellar atrophy	Significant cerebellar atrophy
Muscle biopsy	Not carried out	COX negative fibres, type 2 fibre atrophy, subtle mitochondrial rearrangement, no myositis, dystrophy, degeneration	Not carried out	Not carried out
Nerve Conduction Study	Normal	Normal	Minimal large fibre peripheral neuropathy	Normal
SCA 1,2,3,6 gene	Negative	Negative	Negative	Negative
Other tests- discussed during the	Negative Episodic ataxia -2, friedreich’s ataxia Normal CSF, -ve OCB	Glycosaminoglycan screen –ve, quantitative amino acid –N, Organic acid in urine-normal	Frataxin Negative, negative anti Glutamatic acid decarboxylase	Negative Frataxin, Fragile X syndrome, negative SCA 7 and 17

The Magnetic resonance imaging (MRI) showed some degree of cerebellar atrophy in first three patients. However the last patient had significant pan-cerebellar atrophy in MRI scan (**Table 2**). None of them showed any evidence of cord compression, demyelination or space occupying lesion in whole spine magnetic resonance imaging. The genetic screening for spinocerebellar ataxia (SCA 1,2,3 and 6) were negative for all. Patient 4 had additional testing for mutation in SCA 7 which turned out to be negative.

The next generation sequencing involving 21 genes in the spastic paraparesis panel revealed autosomal recessive hereditary spastic paraparesis due to the mutation in Spastic paraparesis 7 gene (SPG 7) in all of them with a novel mutation in one of them (**Table 3**). All these mutations were comfirmed by conventional Sanger sequencing.

**Table 3:** Outcome of the Spastic Paraparesis 7 (SPG 7) gene study and treatment.

3^rd^ visit	After 6 months of the 2^nd^ visit	After 6 months of the 2^nd^ visit	After 8 months of the 2^nd^ visit	After 5 months of the 2^nd^ visit
SPG 7 Gene study result discussion	homozygous mutation in exon 11 of the SPG7 gene (c1529C>T pAla510Val)	compound heterozygous mutation (exon 12,14) of c1529C>T pAla510Val and c1672A>T p(Lys558)	Heterozygous c1529c>T, p(Ala510Val) mutation in exon 11 and the c1617delC, p(Val540fs) frameshift mutation in exon 12	Heterozygous c1529c>T, p(Ala510Val) mutation in exon 11 and the c1672A>T, p(Lys 558) mutation in exon 13 of the SPG 7 gene
Outcome and treatment	Regular follow up in 6 months, Baclofen, Physiotherapy	Regular follow up in 12 months, Baclofen, Physiotherapy	Regular follow up in 12 months, Physiotherapy	Transferred to general practice on patient’s request, physiotherapy

### Therapeutic intervention

All the patients were informed approximately their results and were sent for physiotherapy and occupational therapy assessment. The first 2 were also prescribed Baclofen for spasticity.

### Follow up and outcomes

Subsequently during the follow up the first 2 patients complained that their balance worsened while the remaining 2 patients said that they were unchanged. The 2 young patients were referred for further genetic counselling as they were concerned about their children (**Table 3**).

## Discussion

**Figure 1:**
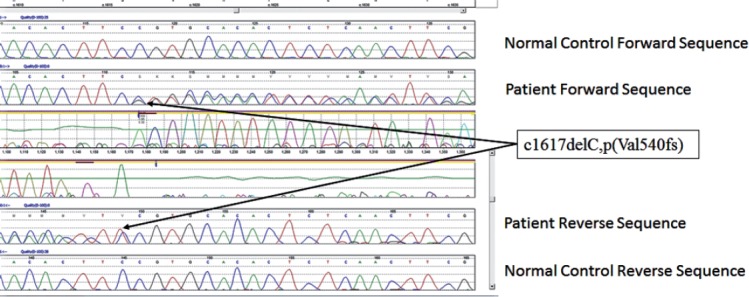
Novel heterozygous pathogenic mutation in the SPG 7 gene, c1617delC, p(Val540fs) with the Sanger sequencing confirmation of the c1617delC, p(Val540fs) mutation as viewed in Mutation Surveyor.

**Figure 2:**
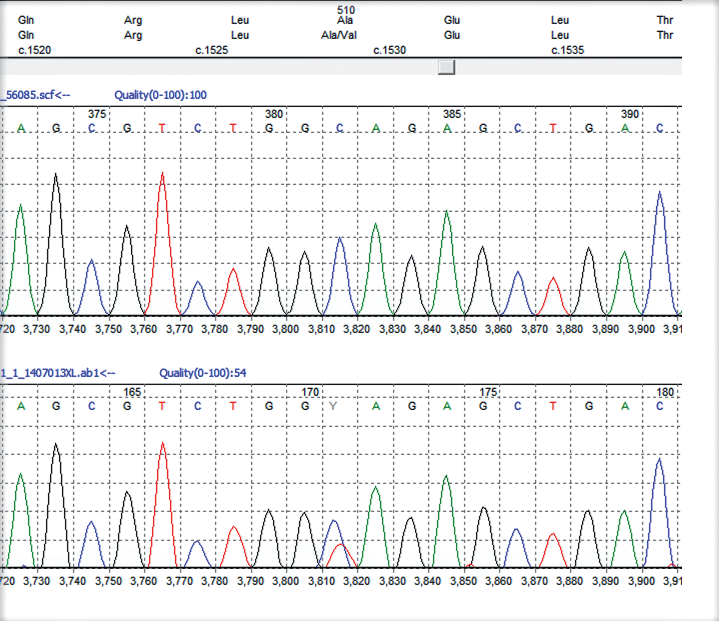
Heterozygous c1529c>T, p(Ala510Val) mutation in exon 11 and the c1672A>T, p(Lys 558) mutation in exon 13 of the SPG 7 gene (The top trace in each diagram is the normal control sequence with the patient trace below).

All patients stated that they were struggling to cope with the symptoms but they were relieved to have a diagnosis though not treatable. First 2 pateints still work but mostly office jobs. In our series of 4 patients all had ataxia with 3 had ophthalmoplegia of variable degrees. One had a novel mutation (c1617delC, p(Val540fs) in exon 12 (**Figure 1**). Since the mutation created a frameshift it is highly likely to be pathogenic though we could not study the full family. The variant pAla510 Val was the most common mutation in the SPG 7 gene (**Figure 2**). The common exons of the SPG 7 gene involved in the mutation process were 11 and 12. Out of the 77 different mutations of SPG7 gene that have been identified so far 9 were gross deletions.Warnecke T et al^6^ found a new missense mutation c.2075G>C in exon 15 of the SPG7 gene in the homozygous state, substituting serine with threonine at codon 692. Sanchez Ferrero et al^7^ reported 12 new mutations in SPG 7 gene among the Spanish families. Of these 12 new mutations 5 affected the paraplegin function, 4 non sense mutations, 2 gross deletions etc. The SPG deletion was rarely reported in the literature but this study found nearly 14% deletions. Sanchez Ferrero et al^7^ also clearly showed that career pAla510val variant was more frequent in patients vs healthy controls (3% vs1%). Elluech N et al^1^ reported 47 genetic variations including 6 mutations, 27 polymorphisms etc. Klebe S et al showed that pAla51oVal mutation was the commonest mutation of SPG 7 gene.^8^ They also reported a novel missesne SPG 7 mutation (Asp411Ala) which resulted in Autosomal Dominant optic Neuropathy in a SPG 7 mutant family. Pfeffer et al found novel mutation in 3 persons and pointed out that SPG 7 mutation could cause progressive ophthalmoplegia through disordered mitochondrial maintenance.^9^ Van Gassen et al discovered 14 previously unreported mutations.^10^ SPG 7 encodes ‘paraplegin’, a mitochondrial AAA metalloprotease which works closely with another protein AFG3L2. 2Both paraplegin and AFG3L2 are highly expressed in Purkinje fibres of the cerebelleum.^4^ This explains why ataxia and ophthalmoparesis (due to mitochondrial dysfunction) are common in SPG7 mutation. In fact, our series shows that ophthalmological signs are important diagnostic clue for the SPG 7 genetic testing especially when the next generation sequencing not affordable or widely available.

AAA metalloproteases of the inner mitochondrial membrane, paraplegin and AFG3L2, participate in the biogenesis and maintenance of the mitochondrial respiratory chain complexes.^2^ Paraplegin deficiency in HSP does not result in the loss of m-AAA metalloprotease activity in brain mitochondria. The formation of m-AAA metaloproteases with altered substrate specificities probably leads to leads to axonal degeneration in HSP.^2^ Pfefer G et al^4^ showed SPG7 mutations caused increased mitochondrial biogenesis in muscle, with the clonal expansion of mitochondrial DNA mutations resulting in eye symptoms and myopathy.^9^ There are only few studies to show whether clinical features mitochondrial dysfunction secondary to SPG 7 mutation becomes more prominent with growing age and if any phenotype genotype correlation exists or not. The complex phenotype of the SPG7 is associated with upper limb involvement, optic nerve involvement and cognitive deficit.^6^ VanGassen Kl et al showed that a null mutation was associated with the co occurrence of cerebellar ataxia, a missense mutation of exon 10 resulted in predominant optic nerve atrophy.^10^ However our cohort was too small to draw any such conclusion.

In conclusion, SPG 7 remains an important causes of undiagnosed ataxia.
